# Site- and electroencephalogram-frequency–specific effects of 800-nm prefrontal transcranial photobiomodulation on electroencephalogram global network topology in young adults

**DOI:** 10.1117/1.NPh.12.1.015011

**Published:** 2025-02-27

**Authors:** Shu Kang, Lin Li, Sadra Shahdadian, Anqi Wu, Hanli Liu

**Affiliations:** a University of Texas at Arlington, Bioengineering Department, Arlington, Texas, United States; b University of North Texas, Department of Biomedical Engineering, Denton, Texas, United States; c Neuroscience Research Center, Cook Children’s Health Care System, Fort Worth, Texas, United States

**Keywords:** transcranial photobiomodulation, tPBM, electroencephalography, EEG, functional brain networks, graph theory, global topographical metrics, graphical topology

## Abstract

**Significance:**

Transcranial photobiomodulation (tPBM) is an optical intervention that effectively enhances human cognition. However, limited studies have reported the effects of tPBM on electrophysiological brain networks.

**Aim:**

We aimed to investigate the site- and electroencephalogram (EEG)-frequency–specific effects of 800-nm prefrontal tPBM on the EEG global network topology of the human brain, so a better understanding of how tPBM alters EEG brain networks can be achieved.

**Approach:**

A total of 26 healthy young adults participated in the study, with multiple visits when either active or sham tPBM interventions were delivered to either the left or right forehead. A 19-channel EEG cap recorded the time series before and after the 8-min tPBM/sham. We used graph theory analysis (GTA) and formulated adjacency matrices in five frequency bands, followed by quantification of normalized changes in GTA-based global topographical metrics induced by the respective left and right tPBM/sham interventions.

**Results:**

Statistical analysis indicated that the effects of 800-nm prefrontal tPBM on the EEG global topological networks are both site- and EEG-frequency–dependent. Specifically, our results demonstrated that the left 800-nm tPBM primarily enhanced the alpha network efficiency and information transmission, whereas the right 800-nm tPBM augmented the clustering ability of the EEG topological networks and improved the formation of small-worldness of the beta waves across the entire brain.

**Conclusions:**

The study concluded that 800-nm prefrontal tPBM can enhance global connectivity patterns and information transmission in the human brain, with effects that are site- and EEG-frequency–specific. To further confirm and better understand these findings, future research should correlate post-tPBM cognitive assessments with EEG network analysis.

## 1 Introduction

Using near-infrared or infrared light (660 to 1070 nm), transcranial photobiomodulation (tPBM) has shown the potential to improve human cognition, including attention, memory, and executive function in both healthy humans and patients with neurological diseases.^1^^–^^7^ One underlying principle of tPBM is based on light absorption by cytochrome c oxidase (CCO), the main intracellular light-absorbing molecule within the mitochondria. Oxidized CCO in the redox state of the mitochondria has a shallow absorption peak between 800 and 850 nm in the near-infrared (NIR) region, which allows light to penetrate deeper into the tissue. This photonics–bioenergetics mechanism results in unique metabolic effects with benefits for neural enhancement and neuroprotection.^8^^–^^10^ Thus, tPBM has great potential as an interventional tool for treating a variety of neurological diseases. To quantitatively support the underlying mechanism of action of tPBM, our group has reported that tPBM using a 1064-nm laser can increase cerebral blood flow, oxygenation, and metabolic activity in targeted brain regions,^11^^–^^13^ which is consistent with an independent study.^14^ Specifically, we showed that laser tPBM induced significantly prefrontal^15^^,^^16^ and whole-brain^17^^,^^18^ hemodynamic and metabolic connectivity. These experimental observations are well explained by and matched with the accepted mechanism of action of tPBM.^1^^,^^2^

However, research on how tPBM modulates electrophysiological activity and corresponding networks in the human brain is very limited, with only 10 publications published by March 2023 in the literature, according to a recent review paper.^19^ These studies employed multichannel electroencephalogram (EEG) systems with different numbers of electrodes to investigate electrophysiological responses to tPBM at one of four NIR wavelengths (i.e., 810, 830, 850, and 1064 nm). The stimulation setting parameters and locations were case-dependent. Although most of these studies quantified tPBM-induced topographic alterations in EEG power at different frequency bands (delta: 1 to 4 Hz; theta: 4 to 8 Hz; alpha: 8 to 13 Hz; beta: 13 to 30 Hz; and gamma: 30 to 55 Hz), 4 out of 10 also analyzed tPBM-induced modulations in EEG brain networks using either graph theory analysis^20^^–^^22^ or a combination of group singular value decomposition with exact low resolution brain electromagnetic tomography (eLORETA), which is a linear inverse solution method to reconstruct cortical electrical activity with correct localization from the scalp EEG data.^23^ Thus, the existing literature is extremely inadequate for exploring or surveying the site-dependent effects of tPBM on EEG networks.

Therefore, this study aimed to investigate and quantify the site- and frequency-specific effects of 800-nm prefrontal tPBM on EEG network topology in young adults (n=26) by comparing 19-channel wireless dry EEG measurements before and after 8 min of stimulation. The reason for choosing an 800-nm tPBM in this study was twofold. First, either laser or LED lights at 800 to 850 nm have been used more commonly than those at 1064 nm in both preclinical and clinical research.^1^^,^^7^^,^^24^^–^^27^ Thus, it is helpful to further understand EEG global network changes induced by tPBM in this wavelength range. Second, the results in response to 800-nm tPBM would be more novel and intriguing because frequency-specific effects of 1064-nm prefrontal tPBM on EEG power and network topology in young adults were reported already in several of our publications.^22^^,^^28^^,^^29^

Detailed information on the experimental setting, protocols, and methods for this study is given in Sec. 2. In the data analysis, we employed graph theory analysis (GTA)^30^^,^^31^ to quantify changes in EEG-derived network metrics before and after 800-nm prefrontal tPBM on each side of the forehead. GTA is a method that represents the brain as a network of interconnected nodes (i.e., brain regions) and edges (i.e., connections among the brain regions). This approach enables intricacies of connections and communication patterns within the brain.^30^

## 2 Materials and Methods

### 2.1 Subjects

Subjects were considered eligible if they met the inclusion criteria, which included healthy and pain-free individuals who were 18 years of age or older, irrespective of sex or ethnic background, and had the ability to provide informed consent. The exclusion criteria were as follows: (1) individuals with serious medical conditions, including neurological or psychiatric diseases; (2) those with a history of brain injuries or violent behavior; (3) those who had been institutionalized or imprisoned; (4) those taking long- or short-term psychiatric or psychological medications; (5) pregnant individuals; (6) smokers or those with diabetes; and (7) those who were unable to provide informed consent. The Institutional Review Board (IRB) of the University of Texas at Arlington (UTA) approved all experimental procedures.

Thirty-one healthy subjects were recruited from UTA. Because of the high sensitivity of wireless dry EEG to motion artifacts, five participants with excessive motion artifacts during one or more experiments were excluded from the analysis. After exclusion, 26 young healthy humans (14 males and 12 females; age=22.4±2.3 years) participated in five visits of measurements separated by at least 7 days. The IRB of UTA approved all experimental procedures. All measurements were conducted with informed consent from each participant in accordance with the guidelines approved by the IRB of UTA.

### 2.2 Experimental Setup and Protocol of tPBM with Dual-Mode Measurements

**Fig. 1:**
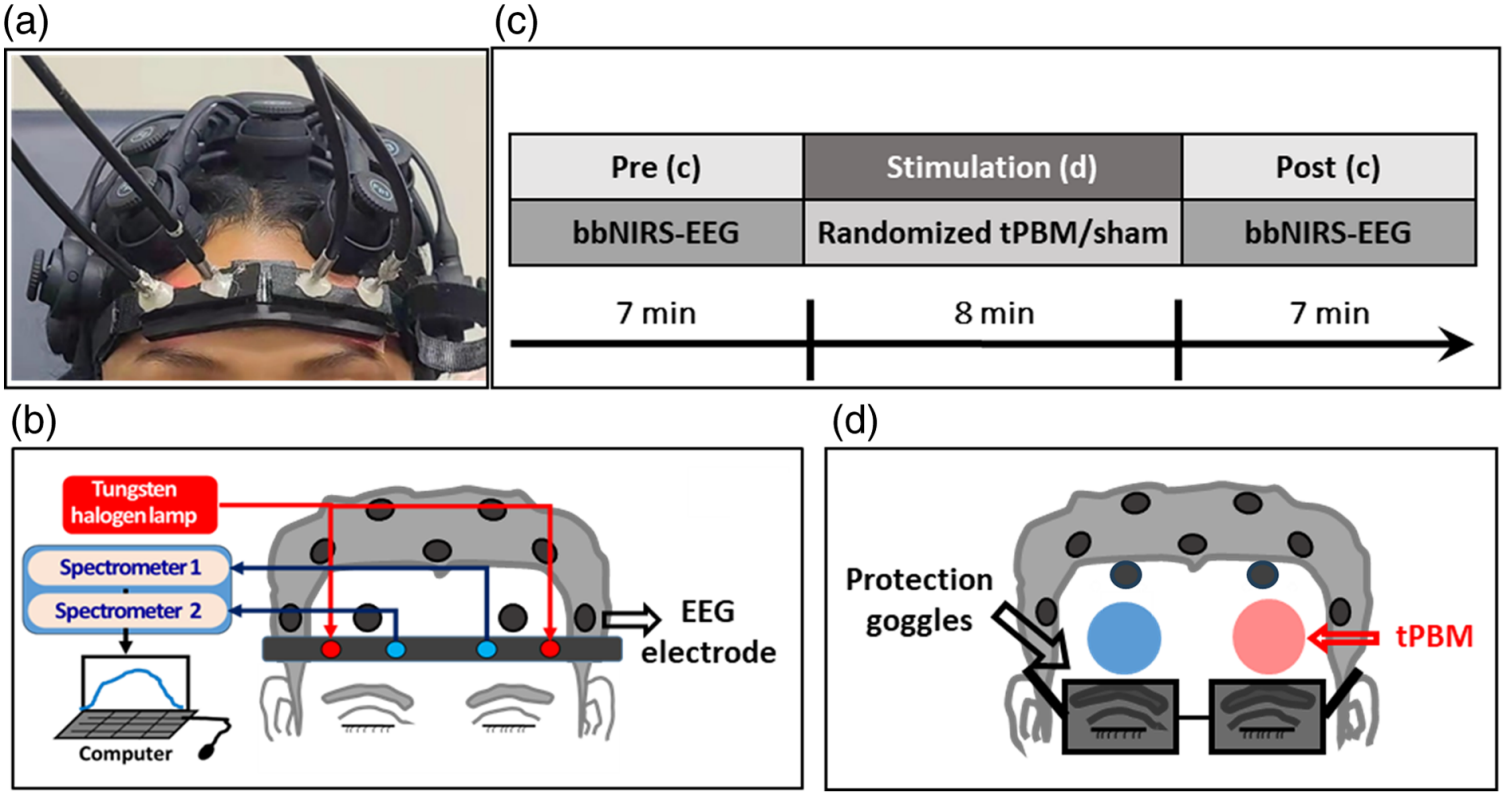
(a) Photo showing the experiment setup of a two-channel bbNIRS on the lateral forehead and a 19-channel EEG cap of a participant. (b) Schematic diagram illustrating the two-channel bbNIRS and the EEG setup. The former is not the interest of this paper; detailed information can be found in Refs. 16 and 32. (c) Experimental protocol of this study; it consists of 7-min eyes-closed pre-stimulation at rest, followed by 8 min of randomized tPBM or sham stimulation on the left forehead (L800), right forehead (R800), left sham (LS), or right sham (RS) in four of the five visits and 7-min post-stimulation. (d) Locations of the tPBM/sham stimulation delivered on the left (red circle) or right (blue circle) forehead.^32^ The bbNIRS holder and EEG channel Fp1 or Fp2 were removed during the left or right tPBM stimulation.

The experimental setup and protocol of the study have been recently reported in Refs. 16 and 32, which focused on alterations in the hemodynamic and metabolic connectivity of the prefrontal cortex in response to prefrontal tPBM. In contrast, this study focused on tPBM-induced modulations in EEG network topology under the same stimulation protocol. For the convenience of the reader, we briefly describe the experimental setup and protocol. As the overall project was designed and carried out for a dual-mode investigation of cerebral responses to tPBM, both 2-channel broadband near-infrared spectroscopy (bbNIRS) and 19-channel EEG were positioned on each subject’s head for concurrent measurements during the pre- and post-tPBM phases [Figs. 1(a) and 1(b)].^32^ The 19-channel EEG consisted of 19 wireless dry electrodes (CGX Quick-20 10–20 EEG system).

In four out of five visits, each participant was randomly assigned to receive left prefrontal 800-nm tPBM (L800), right prefrontal 800-nm tPBM (R800), left prefrontal sham (LS), or right prefrontal sham (RS) stimulation. Each visit was separated from the others by at least 7 days to avoid carry-over effects. During each visit, the experiment lasted 22 min, including a 7-min pre-tPBM (resting state), 8-min tPBM/sham, and 7-min post-tPBM/sham period (resting state), as marked in Fig. 1(c). During the tPBM/sham period (8 min), the headband holding the bbNIRS optodes was detached, whereas the Fp1 and Fp2 electrodes were shifted upward to open the forehead room for light delivery [Fig. 1(d)].

Upon arrival, each participant sat comfortably on a chair, followed by dual-mode probe placement on the participant’s head. After the 7-min baseline measurement, the active tPBM was delivered via 800-nm laser illumination targeted at the left or right frontal region with a light irradiance of $∼250\;\mathrm{mW}/{\mathrm{cm}}^{2}$, whereas the sham was performed with the laser power set to be 0.1 W and the laser aperture covered with a black cap. During tPBM/sham stimulation, the participants were asked to wear protective goggles. The research was conducted using a single-blind crossover design, wherein every participant underwent sham tPBM and sham experiments in a random sequence.

### 2.3 EEG Data Acquisition and Preprocessing

**Fig. 2:**
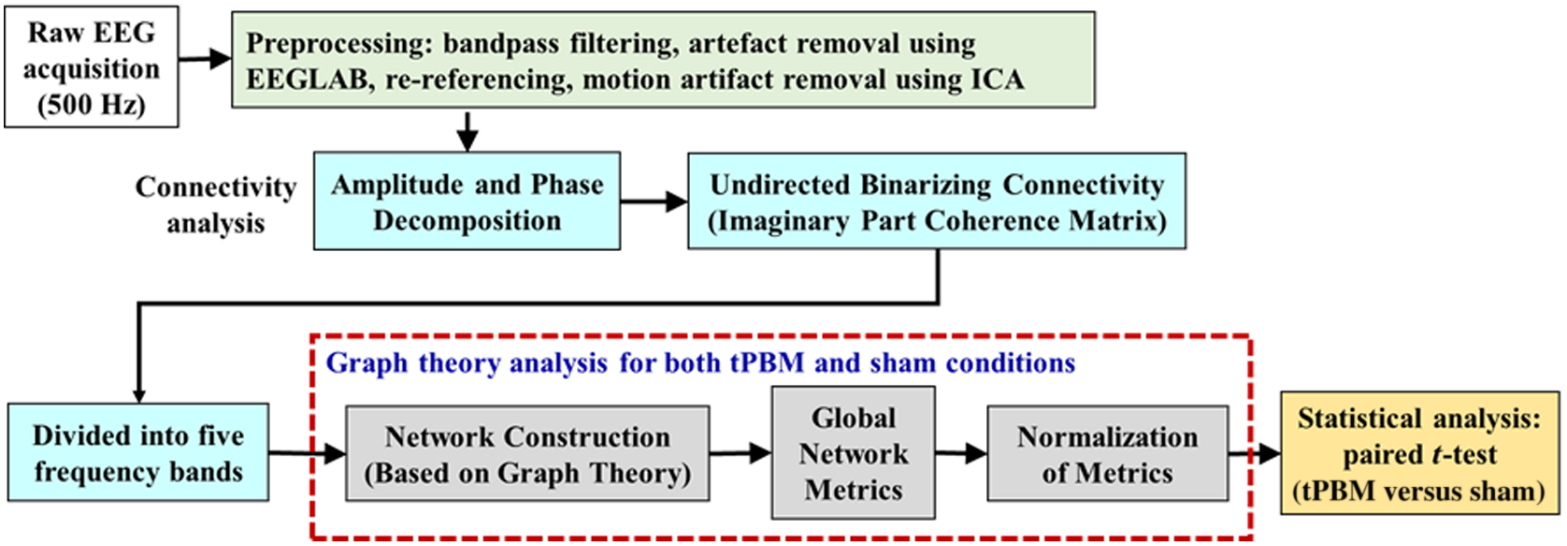
Flowchart of EEG data processing, consisting of steps for (1) pre-processing (green box); (2) amplitude and phase decomposition and the formation of adjacency matrices in five frequency bands (blue boxes); (3) quantification of GTA analysis for both tPBM and sham conditions, which includes network construction, determination of network metrics, and normalization for selected metrics with respect to those before tPBM (gray boxes); and (4) statistical analysis between tPBM and sham stimulations (yellow box).

During the 7-min pre- and post-tPBM/sham periods, the 19-channel EEG (and bbNIRS) recorded electrophysiological signals at a sampling rate of 500 Hz, whereas the participant maintained a state of eyes-closed wakefulness. As depicted by the green box in Fig. 2, all 19-channel EEG signal pre-processing steps were executed using MATLAB^®^ (MathWorks 2022a, MathWorks Inc., Natick, Massachusetts, United States). The raw EEG data sampled at 500 Hz were subjected to bandpass filtering in the range of 1 to 55 Hz using a Butterworth filter. Artificial spikes and flat segments were replaced with combined (50% to 50%) signals from the preceding and subsequent recordings, respectively. Subsequently, the identified channels with large noise or artifacts were removed and replaced by signals that were interpolated from neighboring channels using EEGLAB, an open-source MATLAB toolbox. For each channel of the EEG series, re-referencing was conducted with respect to the averaged EEG signal across all 19 channels. Following this, independent component analysis was applied to decompose the 19-channel EEG signals into underlying independent neural sources, aiming to eliminate motion artifacts, such as eye movements and muscle activity.

### 2.4 Quantification of tPBM-Induced Changes in EEG Network Topology

As outlined in Fig. 2, after pre-processing, each time series of all 19 electrodes was decomposed into amplitude and phase, followed by calculations of the imaginary part of the coherence between each pair of the 19 channels. These coherence values were frequency-dependent and grouped into five frequency bands, followed by the formation of adjacency matrices among all 19×19 electrode pairs. Next, we utilized GTA to quantify tPBM-induced alterations in EEG network topology by determining global graphical metrics. The details of each step are as follows.

#### 2.4.1 Amplitude and phase decompositions (all 19 channels)

For EEG connectivity quantification, correlations among the phases or amplitudes of all EEG channels are used to express functional connectivity among respective electrode sites.^33^^,^^34^ To avoid the volume conduction issue, we performed phase decompositions of the time series for all the 19 channels and represent the amplitude and phase of an EEG signal by a complex number.^34^^,^^35^ Moreover, we utilized multiple tapers (i.e., Slepian sequences) to taper the EEG signal in the time domain before performing the Fourier transform.^36^^,^^37^ This part of the calculation was conducted using the “ft_freqanalysis” function within the FieldTrip toolbox.^38^^,^^39^

#### 2.4.2 Determination of the imaginary part of coherence

To measure connectivity, coherence is commonly utilized in the frequency domain as an equivalent to the time-domain cross-correlation function. The coherence coefficient is computed for a frequency of ω and yields a normalized coefficient between 0 and 1

$${\mathrm{Coh}}_{xy}(\omega )=\frac{\left|S_{xy}(\omega )\right|}{\sqrt{S_{xx}(\omega )S_{yy}(\omega )}},$$ (1)

where $S_{xx}$, $S_{yy}$, and $S_{xy}$ were calculated using complex values obtained with the multitaper method, where $S_{xx}$ and $S_{yy}$ respectively represent the power estimates of signals x and y, and $S_{xy}$ denotes the averaged cross-spectral density term of these two signals.

**Fig. 3:**
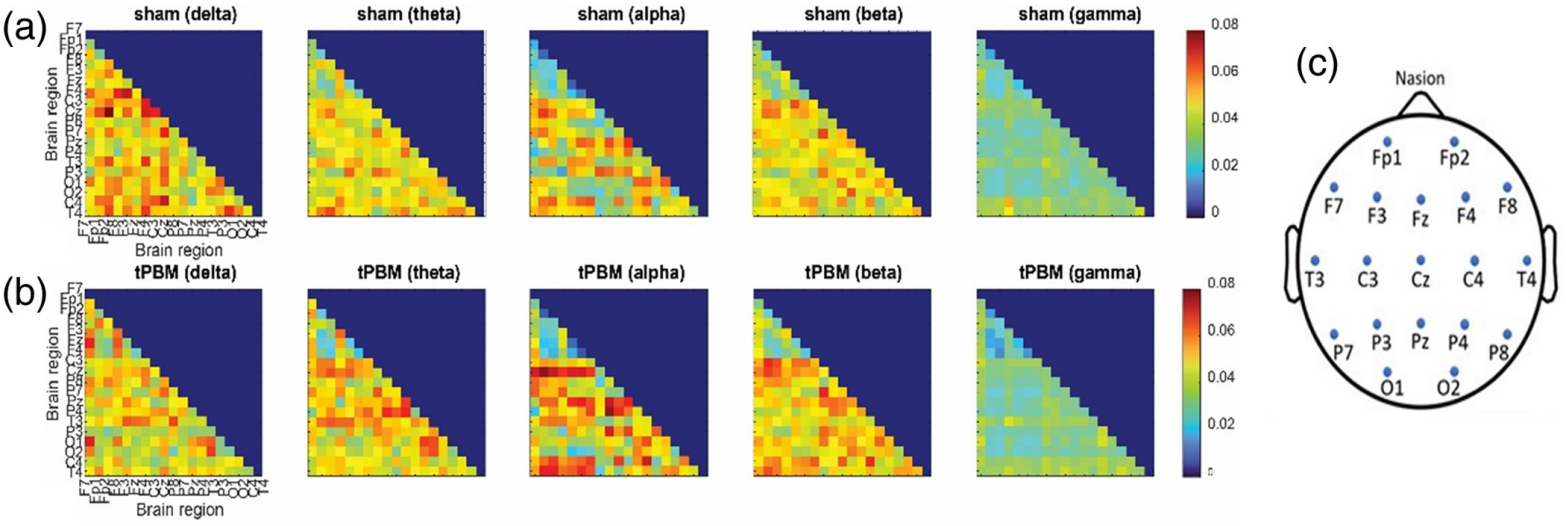
EEG adjacency matrices representing cerebral graphical networks in delta, theta, alpha, beta, and gamma bands under (a) sham and (b) 800-nm left tPBM conditions, averaged over n=26 young adults. (c) Layout of the 19 electrodes used in the study.

To minimize the volume conduction issue, the imaginary part of coherence was used in our study by removing the magnitude operation from Eq. (1) and considering the imaginary part of $S_{xy}$ while setting the cross-spectral density of the signals with 0 or 2π phase difference to zero.^22^ Hence, the “ft_connectivityanalysis” function from the FieldTrip toolbox was utilized for computing the imaginary part of coherence for all pairs of channels. In this study, the pairwise connectivity values for all pairs of electrodes were represented by a 19×19 adjacency matrix. As there was no directional difference between the two coherence values for a pair of electrodes, the adjacency matrix would be symmetrical along the diagonal line and have a total of elements of 19×18/2=171 (Fig. 3).

#### 2.4.3 Subgrouping of coherence values in each of the five EEG frequency bands

For the target temporal segments (i.e., pre- or post-tPBM/sham), each EEG time series was divided into 10-s epochs. For all 19 electrodes, the generated adjacency matrices (i.e., coherence values among different pairs of EEG electrodes) in each frequency band were averaged across all epochs.^22^ These matrices were grouped into five different EEG frequency bands, which were used to obtain GTA-based global connectivity metrics. The five frequency bands include delta (1 to 4 Hz), theta (4 to 8 Hz), alpha (8 to 13 Hz), beta (13 to 30 Hz), and gamma band (30 to 55 Hz). This operation was carried out for each participant’s EEG data.

#### 2.4.4 GTA-based quantification of tPBM-induced changes in global graphical metrics

Graph theory analysis is a powerful and convenient tool^40^ that can help identify the topological features of brain networks. Major global network metrics include (1) small-world properties (clustering coefficient $C_{p}$, characteristic path length $L_{p}$, normalized clustering coefficient γ, normalized characteristic path length $L_{p}$, and small-world σ) and (2) efficiency parameters (local efficiency $E_{\mathrm{loc}}$ and global efficiency $E_{g}$).^31^ Several studies have reported the feasibility of using GTA to analyze EEG time series and consequently to determine the effects of tPBM on brain network metrics.^20^^,^^41^ Our group has also applied GTA-based network analysis to a 64-channel time series for each of the five EEG oscillation frequencies to investigate tPBM effects, as reported in Ref. 22.

A user-friendly graph theory toolbox, GRETNA, was used to quantify the global graphical metrics of brain networks across five frequency bands.^42^ This procedure was repeated 19 times to assess the selected metrics within a sparsity range of 5% to 95%, with a step length of 5%. Regarding the global network metrics, we focused on several small-world properties, such as $C_{p}$, γ, small-world σ, and $L_{p}$, and the network local/global efficiency, $E_{\mathrm{loc}}$ and $E_{g}$.^43^

To investigate tPBM-induced changes in graphical metrics, we normalized the metrics with respect to their own pre-tPBM values, as defined by

$$M_{\mathrm{nor}}^{\mathrm{tPBM}}=\frac{M_{\text{post}}^{\mathrm{tPBM}}-M_{\mathrm{pre}}^{\mathrm{tPBM}}}{M_{\mathrm{pre}}^{\mathrm{tPBM}}},$$ (2a)

$$M_{\mathrm{nor}}^{\mathrm{sham}}=\frac{M_{\text{post}}^{\mathrm{sham}}-M_{\mathrm{pre}}^{\mathrm{sham}}}{M_{\mathrm{pre}}^{\mathrm{sham}}},$$ (2b)

where M is a general symbol representing any graphical network metric/parameter; $M_{\mathrm{nor}}^{\mathrm{tPBM}}$ and $M_{\mathrm{nor}}^{\mathrm{sham}}$ indicate the normalized network metrics; $M_{\mathrm{pre}}^{\mathrm{tPBM}}$ and $M_{\text{post}}^{\mathrm{tPBM}}$ represent the network metrics pre- and post-tPBM, respectively; and $M_{\mathrm{pre}}^{\mathrm{sham}}$ and $M_{\mathrm{pre}}^{\mathrm{sham}}$ express the network metrics pre- and post-sham, respectively. Then, a statistical comparison of global networks between tPBM and sham conditions was performed using paired two-sample t-tests for each global graphical measure at every sparsity level.^44^ A criterion of p<0.05 was selected to define statistical significance between the two conditions.

## 3 Results

### 3.1 Frequency-Specific Adjacency Matrices as EEG Graphical Networks Before and After tPBM

To investigate tPBM-induced changes in functional brain networks, we quantified frequency-specific brain networks for all five bands. Specifically, based on the frequency-domain approach, we formed or constructed a 19×19 adjacency matrix by quantifying the imaginary part of coherence based on Eq. (1) under pre-frontal tPBM or sham conditions for each participant. The respective coherence coefficients for 19 pairwise electrodes were frequency-dependent; thus, five sets of group-averaged (n=26) adjacency matrices in the delta, theta, alpha, beta, and gamma bands were obtained under 8-min left tPBM and sham intervention, as shown in Figs. 3(a) and 3(b), with the electrode layout illustrated in Fig. 3(c). Although Fig. 3 provides comprehensive adjacency matrices representing EEG graphical networks in response to the left 800-nm prefrontal tPBM, similar formatted figures can be obtained for the right tPBM intervention.

### 3.2 Frequency-Specific Significant Changes in Normalized Global Graphical Metrics by Left tPBM

**Fig. 4:**
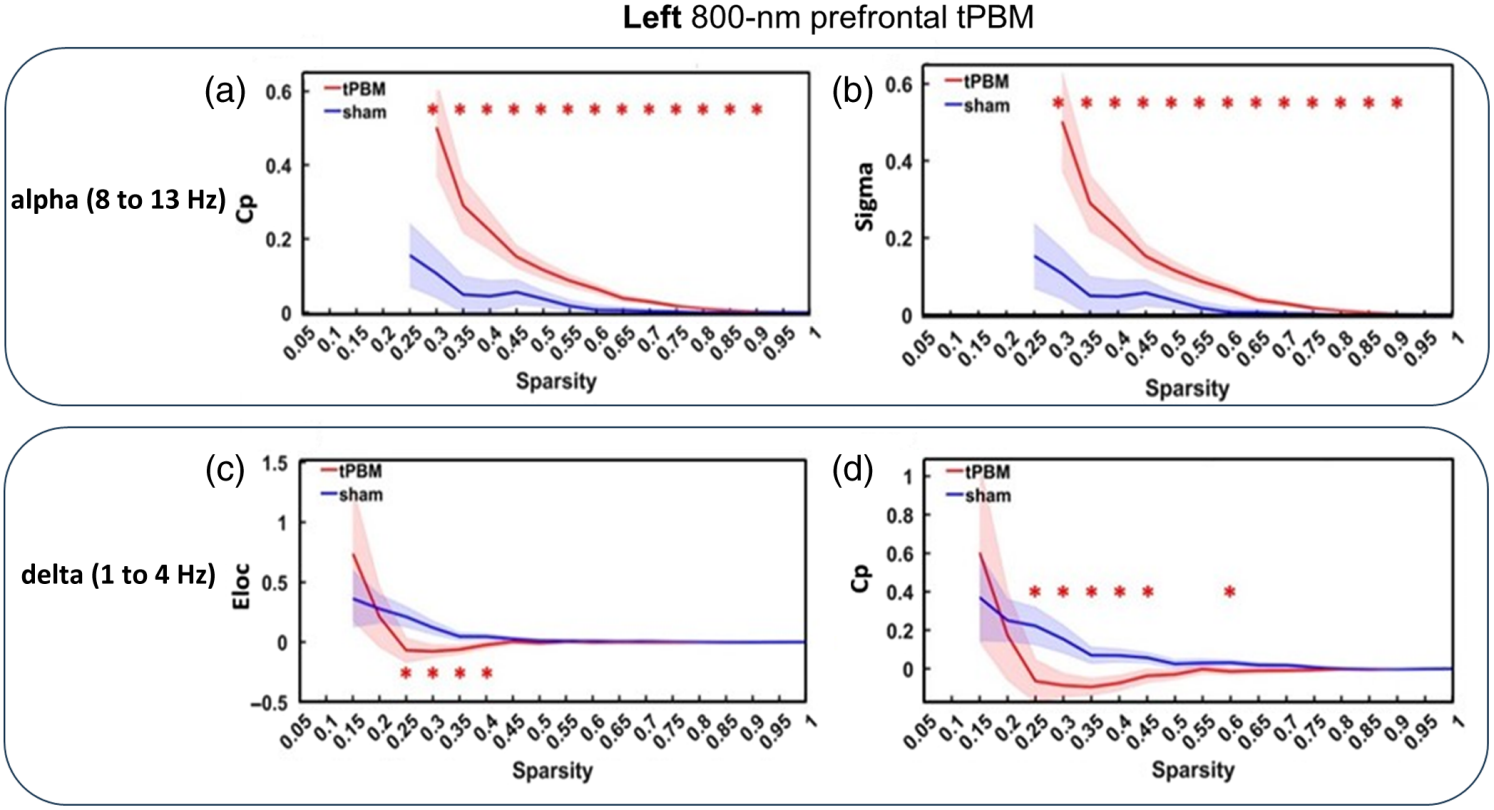
Comparisons among changes in normalized global graphical metrics at the group level (n=26) between 800-nm left tPBM (red) and sham conditions (blue). The graphical metrics include (a) clustering coefficient, $C_{p}$, in the EEG alpha band (8 to 13 Hz); (b) small-world sigma, σ, in the alpha band; (c) local efficiency, $E_{\mathrm{loc}}$, in the delta band (1 to 4 Hz); and (d) $C_{p}$ in the delta band. Significant differences (p<0.05) between tPBM and sham conditions at each sparsity are marked with “*” after two-sample paired t-tests.

After obtaining five frequency-specific adjacency matrices under both sham and tPBM conditions, a software package, GRNTNA,^42^ was utilized to quantify several key global graphical matrices^45^ in both intervention cases, separately. After normalizing the respective metrics with respect to their pre-tPBM values, as expressed in Eqs. 2(a) and 2(b), we calculated changes in the clustering coefficient, $C_{p}$, and small-word sigma, σ, in the alpha band (8 to 13 Hz), as shown in Figs. 4(a) and 4(b), respectively, under both stimulation and sham conditions within a sparsity range of 0.3 to 0.9. After the statistical comparison, we reported that the left tPBM enhanced significantly both $C_{p}$ and σ in a wide range of sparsity in the alpha oscillation. On the other hand, Figs. 4(c) and 4(d) illustrate that in the delta band (1 to 4 Hz), the left tPBM significantly lowered the effective local efficiency, $E_{\mathrm{loc}}$, in a sparsity range of 0.25 to 0.4, and Cp in the sparsity of 0.25 to 0.45.

### 3.3 Frequency-Specific Significant Changes in Normalized Global Graphical Metrics by Right tPBM

**Fig. 5:**
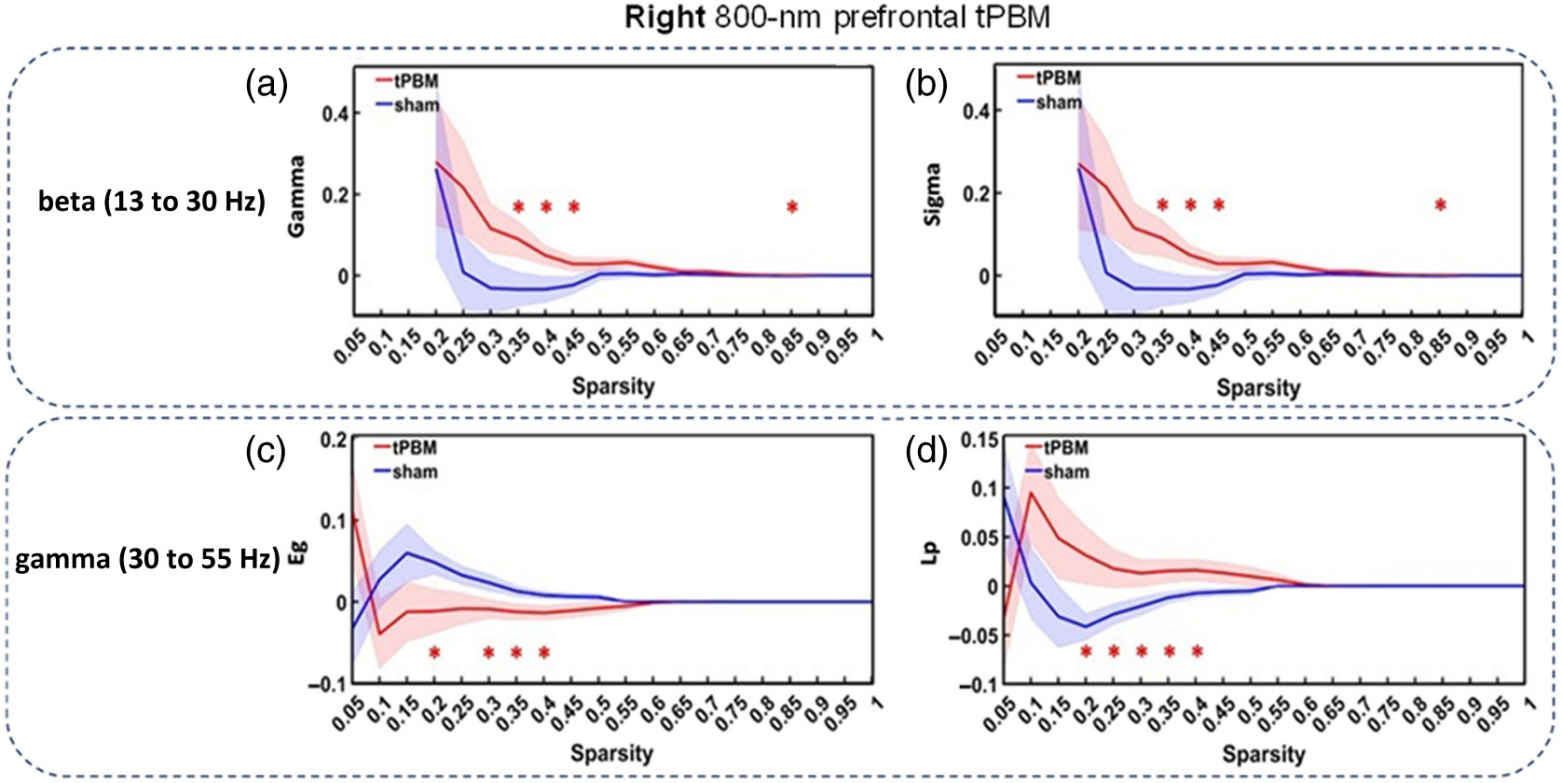
Comparisons among changes in normalized global graphical metrics at the group level (n=26) between 800-nm right tPBM (red) and sham conditions (blue). The graphical metrics include (a) gamma (normalized clustering coefficient), γ, in the EEG beta band (13 to 30 Hz); (b) small-world σ in the EEG beta band; (c) global efficiency, $E_{g}$, in the EEG gamma band (30 to 55 Hz); and (d) shortest path length, $L_{p}$, in the EEG gamma band (30 to 55 Hz). Significant differences (p<0.05) between the tPBM and sham conditions at each sparsity are marked with “*” after two-sample paired t-tests.

Following analysis steps similar to those used for the left tPBM, we obtained right tPBM-induced changes in several normalized global graphical metrics [see Eqs. 2(a) and 2(b)]. Specifically, Figs. 5(a) and 5(b) illustrate the normalized increases by the right tPBM in the normalized clustering coefficient, γ, and small-world σ, within the sparsity range of 0.35 to 0.45 in the EEG beta band (13 to 30 Hz). Furthermore, in the EEG gamma (30 to 55 Hz) band, 800-nm right tPBM significantly lowered the global efficiency, $E_{g}$, of the brain networks in the sparsity range of 0.3 to 0.4 [Fig. 5(c)] and lengthened the shortest path length, $L_{p}$, within the sparsity range of 0.2 to 0.4 [Fig. 5(d)].

Table 1 summarizes the global graphical metrics that were significantly affected by the EEG oscillation frequency bands by either the left or right 800-nm tPBM. No other metrics were found that showed significant differences in the EEG global topological networks between the active and sham 800-nm prefrontal tPBM. One consistent observation from Table 1 is that either left or right 800-nm tPBM induced significant increases in cluster coefficients (either $C_{p}$ or γ) and small-world σ in the EEG alpha or beta bands. Furthermore, graphical parameters in other EEG frequency bands were reduced or had no effects by either tPBM.

**Table 1:** List of global graphical metrics affected by left or right 800-nm tPBM.

EEG frequency band	Left 800-nm tPBM	Right 800-nm tPBM
Delta (1 to 4 Hz)	$E_{\mathrm{loc}}$ ↓; $C_{p}$ ↓ (sparsity of 0.25 to 0.4)	
Theta (4 to 8 Hz)		
Alpha (8 to 13 Hz)	$C_{p}$ ↑; σ ↑ (sparsity of 0.3 to 0.9)	
Beta (13 to 30 Hz)		γ ↑; σ ↑ (sparsity of 0.35 to 0.45 and 0.85)
Gamma (30 to 55 Hz)		$E_{g}$ ↓; $L_{p}$ ↑ (sparsity of 0.2 to 0.4)

*Note*: ↑ or ↓ signify a significant increase or decrease of the marked parameter by the respective 800-nm tPBM.

## 4 Discussion

Although tPBM has shown promising benefits as a useful intervention tool for treating certain neurological diseases, a good understanding of how it alters or modulates the electrophysiological network in the human brain is limited. Previous studies by Wang et al.^28^^,^^29^ from our group have shown that tPBM using a 1064-nm laser can increase alpha and beta rhythms in the frontal and parietal regions during the eyes-open and eyes-closed resting state, as measured in healthy humans using 64-channel EEG. The same human data enabled us to explore the significant effects of the 1064-nm right prefrontal tPBM on EEG-based cerebral connectivity and network topology.^22^^,^^23^ However, the impact of left and right prefrontal tPBM on brain electrophysiological networks with an 800-nm laser has not been investigated. This study addressed this gap with interesting results that provided findings relatively consistent with those of previous reports^22^^,^^23^^,^^28^^,^^29^ and posed new questions for future investigation.

### 4.1 Understanding of GTA-Derived Global Network Metrics in the Human Brain

In GTA,^30^^,^^31^ a small-world network is characterized by a combination of high local clustering and short global path lengths, which enable the brain to process information efficiently, both locally within specific regions and globally across the entire brain. Let us examine several key metrics that were affected by the left or right tPBM in Table 1.

1. $C_{p}$: This is the average of the clustering coefficients over all nodes in a network and quantifies the extent of local cliquishness of a network. A higher $C_{p}$ in a small-world network tends to process information more efficiently.^31^
2. Normalized clustering coefficient γ: This is the ratio of $C_{p}$ between a small-world network and a random network; it must be greater than 1. It assesses the efficiency of information processing and integration within a brain network.^46^ A larger γ indicates a more effective small-worldness.^43^
3. Small-world index σ: This metric summarizes the small-world properties into a simple quantitative small-worldness index. A higher σ implies higher clustering and shorter average path length, thus enhancing local processing and global communication in the human brain.^31^
4. $L_{p}$: This is one of the small-world properties, defined as the average of the shortest path lengths between any pair of nodes in the network.^31^ It measures the efficiency of communication between two nodes in the network.^47^^–^^49^
5. $E_{g}$ and $E_{\mathrm{loc}}$: Global and local efficiency measure the ability of a network to transmit information at the global and local level, respectively.^31^ The bigger they are, the more efficient a network can transmit information.^50^^,^^51^

In summary, for the listed five global graphical metrics, an increase in any of the first three metrics (i.e., $C_{p}$, γ, and σ) is generally beneficial for human brain performance because it enhances both the local and global efficiency of information processing. This optimal network organization supports cognitive functions, learning, and overall brain health. On the other hand, an increase in $L_{p}$ or a decrease in $E_{g}$ and $E_{\mathrm{loc}}$ would reduce the efficiency or operation speed of the human brain.

### 4.2 Enhanced Global Topographical Networks at Alpha and Beta Bands by 800-nm Prefrontal tPBM

Figure 4 and Table 1 clearly illustrate that left 800-nm prefrontal tPBM significantly increased the values of both the clustering coefficient, $C_{p}$, and small-worldness, σ, in the EEG alpha oscillations (8 to 13 Hz) with a broad sparsity range, as compared with the sham stimulation. In the meantime, Fig. 5 and Table 1 demonstrate that the right 800-nm prefrontal tPBM also significantly enhanced the values of the normalized clustering coefficient, γ, and the same σ in the EEG beta (13 to 30 Hz) oscillations. Taken together, these two sets of results suggest consistent and positive effects of 800-nm prefrontal tPBM delivered on either lateral side of the forehead on the enhancement of global topographical networks in the alpha and beta bands. The results of this study imply that left prefrontal tPBM with an 800-nm laser could enhance functional connectivity within the entire cortex in the alpha band.

It has been known that alpha oscillation is linked to states of relaxed wakefulness, attentional processes, and cognitive control.^52^^–^^55^ It has also been reported that alpha and beta rhythms of brain oscillations result from the synchronous electrical activity of thalamocortical neurons, with alpha being more characteristic of quiet awake states and beta of alert states.^56^^,^^57^ More specifically, alpha waves are robustly modulated during cognitive processes and play an important role in the integration and communication among different brain rhythms during brain activity.^58^ Furthermore, beta oscillations are also relevant to cognitive processing but to a lesser degree than alpha waves.^59^^,^^60^ Our results revealed that the right prefrontal tPBM was able to create large effects on the global EEG networks in beta oscillation, which is consistent with our previous studies^22^^,^^28^^,^^29^ and another independent study.^20^ These observations led us to speculate that the right prefrontal tPBM (using either an 800- or a 1064-nm laser) strengthens global EEG beta oscillations across the entire brain with increased clustering ability and small-world effectiveness.

### 4.3 Reduced Global Topographical Networks in Delta and Gamma Bands by 800-nm Prefrontal tPBM

In contrast to the enhancements of the global EEG alpha and beta networks, the left and right 800-nm prefrontal tPBM reduced the global delta and gamma networks, respectively. As shown in Table 1, the left tPBM downgraded the local efficiency, $E_{\mathrm{loc}}$, and cluster coefficient, $C_{p}$, across the entire brain in the delta oscillation (1 to 4 Hz). Meanwhile, the right tPBM also lessened $E_{g}$ and lengthened the path length $L_{p}$ in the gamma band (30 to 55 Hz). Alterations in all four metrics result in less efficient and slower communication and information processing^51^ within the brain networks in the respective oscillation bands.

The delta band (1 to 4 Hz) is associated with deep sleep, restorative processes, and emotional regulation,^61^^,^^62^ whereas gamma waves are often observed during tasks that involve sensory perception, memory formation, and higher-level cognitive functions. A decrease in $C_{p}$ of the small-world network in the delta band indicates a more dispersed network with longer path lengths. This could potentially facilitate collaboration among regions with a greater spatial separation. Furthermore, an increase in $L_{p}$ implies an extension of the overall path length in network communication. Interestingly, the observations of a reduction in $E_{g}$ and an increased $L_{p}$ in the gamma band, implying a decrease in the network efficiency or power, are aligned with the report by Ref. 23, which was analyzed using a completely different method. Taken together, we speculate that left and right 800-nm prefrontal tPBM initiated more complex or dispersed electrophysiological processes, making the paths of information transmission longer and local efficiency lower, in both delta and gamma oscillations.

Focusing on the tPBM-affected beta and gamma oscillations alone (see Table 1), our observations suggest that the right prefrontal 800-nm (and 1064-nm^21^) tPBM enables improvement of network efficiency with a larger clustering coefficient and a more effective small-worldness while creating more dispersed cognitive processes and less global operations.

### 4.4 Site- and EEG-Frequency–Specific Effects of 800-nm Prefrontal tPBM on Global Networks

The current brain research field widely speculates that distinct anatomical structures of the human brain play divergent roles in executing various functions and tasks.^63^^–^^66^ For example, the left frontal region is believed to handle cognitive functions, such as decision-making, planning, and problem-solving. The right centrotemporal area is thought to process auditory information and language comprehension, whereas the left centrotemporal area may be linked to language processing and auditory perception. The right parieto-occipital region manages visual processing, spatial perception, and visual attention. The right frontal region regulates emotions, social interactions, and decision-making. It would be highly desirable and medically useful if a required tPBM beam, used as a disease treatment, can be accurately delivered to the selected or desired brain region(s). To do so, we must understand how and what global and local brain networks are affected by the chosen tPBM protocol, as demonstrated in this study.

Based on the detailed discussions in Secs. 4.2 and 4.3 and Table 1, this study demonstrated that the left 800-nm tPBM primarily enhanced the alpha network efficiency and information transmission, whereas the right 800-nm tPBM augmented the clustering ability of the EEG topological networks and improved the formation of small-worldness of the beta waves across the entire brain. Theoretically, tPBM deliveries in both lateral prefrontal cortices are expected to create the same alterations in EEG topological network metrics, without lateralization. However, this expectation did not match our experimental observations. Because of the small sample size, extremely limited scientific reports in the literature, and several limitations (as listed in Sec. 4.6) of the study, our observations that bilateral prefrontal tPBM stimulations resulted in a differential effect on the oscillatory behavior of the brain need to be further confirmed. However, our results are worth reporting as early-stage scientific evidence.

### 4.5 Justification of the EEG Measurement Setup and Protocol Chosen

The decision to use the current 19-channel dry EEG device was because of its easier, faster, or more convenient setup, as well as its more compact interface, as compared with the 64-channel wet EEG system employed in our previous studies.^22^^,^^28^^,^^29^ The placement of the dry, wireless 19-channel electrodes can be completed in just a few minutes, without the need for delicate wires or tedious application of conductive gel to each electrode tip. In addition, the absence of a tight-fitting cap and reduced setup time make it a practical choice for multi-session studies in both research and clinical settings. However, a potential drawback of dry EEG electrodes is the high risk of electrical noise, which may exceed acceptable levels. A practical objective of this study was to evaluate the feasibility and utility of this device, considering these challenges.

This study focused on global EEG brain networks in the absence of external visual stimuli. Therefore, the eyes-closed condition was more aligned with the experimental objectives. In the eyes-open state, visual stimuli can trigger intense brain activity, particularly in the visual cortex. Eye movements, such as blinking, may introduce artifacts into EEG signals. On the other hand, the eyes-closed state reduces such artifacts, resulting in cleaner EEG signals and facilitating subsequent analysis. Consequently, the eyes-closed condition minimizes visual input interference, allowing for a more focused examination of the intrinsic brain activity.

### 4.6 Limitation of the Study and Future Work

This study has several limitations. First, the EEG cap was moved between pre- and post-tPBM measurements. This was because of the space required on the forehead for tPBM light delivery. This process could cause possible non-alignments of each electrode between the pre- and post-tPBM measurements, leading to position and calculation errors for accurate tPBM effects. Second, the study was performed in an eyes-closed resting state, which might have induced gradual drowsiness in some participants. It is known that the drowsy state could potentially affect the quality of the recorded EEG data, potentially introducing a different electrophysiological state of the EEG signal, which can contaminate the tPBM-related signal. Third, we were unable to obtain consistent findings regarding significant changes in nodal topographical networks by 800-nm prefrontal tPBM. This failure could result from (i) a limited number of (19) electrodes, (ii) larger electrical noise due to dry and wireless electrodes, and (iii) possible inconsistent electrode locations between the pre- and post-measurements. Last, we did not ask the participants to perform any cognitive functions, such as working memory or attention, so we could not directly assess and associate post-tPBM brain connectivity and efficiency with significant improvements in human cognition. To overcome these limitations, we propose that future studies should (1) reduce the electrode sizes at Fp1 and Fp2 and possibly the size of the tPBM light aperture, so there is no need to move any electrode before and after the stimulation; (2) ensure that each participant has a good night sleep the night before the experiment; (3) use 64-channel conventional EEG to increase the number of electrodes and reduce the electrical noise; and (4) include a couple of cognitive tasks pre- and post-tPBM so that we can prove or confirm that tPBM indeed facilitates enhancement of cognitive functions with improvement of brain connectivity and/or efficiency.

## 5 Conclusions

This study investigated the site- and EEG-frequency–specific effects of 800-nm prefrontal tPBM on EEG global network topology in 26 young adults. After careful time-frequency data processing of the recorded 19-channel EEG time series before and after the left and right 8-min tPBM interventions from each participant, we formulated adjacency matrices in five frequency bands and normalized changes in GTA-based global topographical metrics induced by the respective left and right tPBM interventions. Statistical analysis indicated that both left and right 800-nm prefrontal tPBM enabled the significant enhancement of the EEG neurophysiological networks in the alpha and beta oscillations, respectively, and improved human brain processing efficiency. These findings demonstrate that 800-nm prefrontal tPBM can help augment connectivity patterns and information transmission in the human brain, with effects that are site- and EEG-frequency–specific. The observed changes in network metrics may have a close neurophysiological association with tPBM-induced enhancement of human cognition. To further confirm and better understand these findings, future research should correlate post-tPBM cognitive improvement with EEG network analysis.

## Data Availability

The data presented in this study are available on request from the corresponding author.
